# To Stay or to Leave: The Role of School, Family, and Prosocial Goals in Migration Intentions of Russian High School Students

**DOI:** 10.11621/pir.2024.0105

**Published:** 2024-03-15

**Authors:** Tamara O. Gordeeva, Oleg A. Sychev, Dmitry S. Kornienko, Natalia A. Rudnova, Marfa I. Dedyukina

**Affiliations:** a *Lomonosov Moscow State University, Russia*; b *Federal Scientific Center of Psychological and Multidisciplinary Research, Moscow, Russia*; c *Altai State Pedagogical University, Barnaul, Russia*; d M. K. Ammosov North-Eastern Federal University, Yakutsk, Russia

**Keywords:** migration intentions, high school students, school satisfaction, family support, prosocial life goals, subjective well-being, academic achievement

## Abstract

**Background:**

Migration intentions are extremely common among modern youth practically all around the globe. They do not always result in actual migration, but they should definitely be considered as an important indicator for the society, since the potential loss of the most valuable human resources entails long-term consequences for the development of a country or region. This study aims to examine the system of psychological factors that determine the desire of young people to stay in their region. It also addresses the previously discovered paradoxical association between migration intentions and subjective well-being.

**Objective:**

To analyze the psychological determinants of the intention not to leave the home region, and its association with relationship satisfaction, personal goals, subjective well-being, and academic achievement of high school students.

**Design:**

The cross-sectional study design was used. A questionnaire-based survey was conducted among Russian high school students from urban and rural schools (*N* = 5,635).

**Results:**

The study found that the most important psychological predictors of the intention to stay, to study, and work in their home city/region are community contribution goals and psychological factors that characterize the immediate social environment, which include satisfaction with school and teachers, and family support. Controlling for these variables, migration intentions do not correlate with subjective well-being.

**Conclusion:**

These findings suggest that considering the quality of interpersonal relationships allows deepening the understanding of migration intentions sources of high school students. The study also contributes to understanding the complex relationship among migration intentions, subjective well-being, and academic achievement.

## Introduction

In recent decades, there have been active processes of migration within Russia, between regions, republics, and autonomous districts; from remote areas of the country to large cities; between the countries of the former USSR; as well as departure from the country in connection with the beginning of the special military operation (Feb. 24, 2022) (Federal State Statistics Service, 2024). This determines the relevance of this study, which explores the reasons for the desire to stay in one’s native region. The problem of migration is an intricate one, since the intention to migrate, which triggers actual migration, is determined by a complex of various factors and causes. There are several groups of factors that support the desire and intention to migrate and actual migration to another region (internal migration) or to another country (external migration). These factors can be categorized as personality-related, motivational, socio-cultural, economic, and socio-demographic.

Personal and motivational reasons, including various personality traits, values, motives, and states of (dis)satisfaction, have been thoroughly studied by psychologists in recent decades. Individual values as predictors of migration intentions and behavior were of the most immediate interest, both at the theoretical level (Tartakovsky & Schwartz, 2001) and the empirical level (Murashchenkova, 2021; Murashchenkova et al., 2023; Sychev et al., 2021). Some studies explored the association of the intention to migrate, migratory behavior, and successful adaptation in a new region with the Big Five traits (Tabor et al., 2015), persistence (Tabor et al., 2015), academic intrinsic motivation (Rudnova et al., 2023), self-effcacy (Van Dalen & Henkens, 2008), optimism (Berlinschi & Harutyunyan, 2019), civic and ethnic identity (Murashchenkova et al., 2022; Sychev et al., 2021), moral foundations (Sychev et al., 2021), and happiness and subjective well-being (for an overview see Hendriks & Burger, 2020). This latter line of research will be discussed in more detail below, as it forms part of our empirical study.

Social reasons include the individual’s perception of the current social and political situation in the country, and his/her evaluation of it in terms of building a future in comparison with possible alternatives. Research on the vision of the present and future of the country as a factor in the emigration of young people in three members of the Commonwealth of Independence States (CIS) — Russia, Belarus, and Kazakhstan — confirmed that migration intentions and behavior of students from different countries can be predicted by different types of representations (Efremenkova et al., 2023). In case of Russian students, migration intentions were more pronounced among those who viewed the country’s present as short-lived, not free, significant, but at the same time, boring. In fact, the more boring the respondents considered their country’s present situation, the more prone they were to migration behavior. Both migration intentions and behavior depended on the number of acquaintances abroad (Efremenkova et al., 2023).

Economic reasons for migration include seeking a higher quality of life, better career opportunities, better-paying jobs, and a higher income (Lu et al., 2009). The role of socio-demographic characteristics of those intending to migrate (such as age, education, gender, marital status, income, income differences, knowledge of a foreign language, experience of travelling and living abroad, etc.) are also worth mentioning (see Docquier et al., 2014; Graham & Markowitz, 2011; Lu et al., 2009; Otrachshenko & Popova, 2014; Sychev et al., 2021). However, factors of this kind often interact with each other, and therefore, it seems optimal to consider them systemically together with psychological factors. For example, it is known that life satisfaction, as a personal factor of migration intentions, is related to income (Hendriks & Burger, 2020), which belongs to the economic category.

Our analysis of prior research on the most actively studied connections between migration intentions and personality traits, happiness, and subjective well-being provided rather contradictory conclusions. For example, a study conducted in New Zealand found that greater openness to new experiences increased the chances that a participant was planning to move abroad, while greater agreeableness and conscientiousness lowered the odds of a move (Tabor et al., 2015). In another study, high openness to new experience, extraversion, and low agreeableness were shown to increase proneness to migration behavior in the US. That study also showed that high openness and low agreeableness predicted both types of migration (intrastate and interstate), while extraversion predicted only internal migration (Jokela, 2009). Fouarge and colleagues showed that German students with high openness to experience and extraversion and low agreeableness, conscientiousness, and neuroticism were more likely to form migratory intentions than students with the opposite personality traits (Fouarge et al., 2019). In general, it can be concluded that only the trait of openness to experience is a stable and consistent predictor of migration intentions, whereas other Big Five traits demonstrate weak and contradictory patterns of results.

In the context of our study, which is focused on the role of human relationships as a factor in migratory intentions, extraversion and agreeableness are of the greatest interest. Interestingly, despite the generally well-established relationship of these traits with each other, extraversion associated with sociability and talkativeness correlates positively with migration intentions, while agreeableness, which shows interest in other people and a willingness to help them, is inversely associated with migration intentions. Extraversion is the second trait that is assumed to be a predictor of migratory intentions (Fouarge et al., 2019). However, its ambiguity in the context of migratory behavior seems quite natural, since the ability to communicate, make contacts, and establish friendships, which is certainly useful in a new environment, does not necessarily ensure reliable relationships with other people (see, for example, Hotard et al., 1989). Our review of previous findings on the relationship of these traits with migration intentions and migratory behavior demonstrated that a more targeted analysis of the individual’s involvement in favorable relationships with other people, and satisfaction with the quality of these relationships, is required.

The inclusion of happiness as a factor influencing migration intentions derives from the need to comprehend the universal pursuit of happiness and well-being among individuals across diverse countries and regions (Ford et al., 2015). This concept has gained prominence in recent years, suggesting that pursuit of happiness may influence migration intentions and behavior. Correlational evidence does show that less happy people are more likely to have the desire and intention to emigrate, even after controlling for standard predictors of migration such as sociodemographic and economic characteristics, and the presence of friends and acquaintances abroad (Brzozowski & Coniglio, 2021; Cai et al., 2014; Graham & Markowitz, 2011; Lovo, 2014; Migali & Scipioni, 2019; Otrachshenko & Popova, 2014). The tendency of unhappy individuals to migrate is true for both affective and cognitive measures of well-being, and is particularly noticeable in high-income countries. However, it can also be observed in middle-income countries (Cai et al., 2014; Migali & Scipioni, 2019). Linkages appear more complex in low income countries: more unhappy people are more likely to have a desire to migrate but are less likely to take real actions to move abroad (Migali & Scipioni, 2019), apparently because of the material hardship they experience. Consistent with the notion that people seek happiness, those who intend to emigrate consider places with higher average life satisfaction, which remains true after controlling for standard predictors of migration such as macroeconomic environment, distance, language, and share of migrants in the country (Lovo, 2014).

However, as A. Ivlevs (2015) rightly points out, the frequently observed relationship between migration intentions and happiness does not necessarily mean that there is a causal effect of the subjective level of happiness on migration intentions. Furthermore, happiness may even show a temporary drop prior to the planned migration, because this challenging decision implies leaving behind close, reliable, and supportive relationships. On the other hand, dissatisfaction with life (including material, interpersonal, and career dissatisfaction) can indeed be an important motive for migration, prompting people to look elsewhere for better conditions (Otrachshenko & Popova, 2014). It should also be considered that in a number of regions, in particular in Eastern Europe, this association is very weak (Lovo, 2014). The greater desire to migrate among the less happy people results in real migration behavior only in certain groups of the population, such as women and working citizens (Brzozowski & Coniglio, 2021).

We suggest that it is not happiness and life satisfaction as such, but the psychological factors behind them, that determine young people’s desire to leave or stay in the region. These factors primarily include the quality of their significant relationships, i.e., with friends, family, teachers, and classmates. Psychological theories predict (Self-Determination Theory, Ryan & Deci, 2017; PERMA model, Seligman, 2002), and research confirms, that warm and supportive interpersonal relationships are one of the strongest predictors of well-being, happiness, and life satisfaction (Diener et al., 2010). The research data collected among adolescents indicates that happy students tend to report positive relationships with their teachers (Reddy et al., 2003; Suldo et al., 2009), and warm and supportive relationships with their parents (Vansteenkiste & Ryan, 2013).

The significance of interpersonal relationships as a factor in migration intentions and behavior is evidenced by three types of data. The perception of the broad social environment and the feeling of belonging to the local community mitigate the development of migration intentions (Cai et al., 2014). It was also shown that a small number of friends (Frieze et al., 2011; Lu et al., 2009) and connections abroad (Efremenkova et al., 2023; Lu et al., 2009) are important contributors to migration intentions and behavior.

To our knowledge, though, there are no studies focused on the connection of migration intentions and (dis)satisfaction with one’s interpersonal relationships, or between migration intentions and prosocial life goals. We could not locate any research on the role of psychological factors related to migration intentions of high school students, with the exception of one study linking migration intentions with intrinsic and extrinsic academic motivation and alienation from school (Rudnova et al., 2023). Therefore, we put forward a hypothesis that high school students dissatisfied with their relationship with their immediate environment would be more inclined to leave their city/region. These inclinations would be supported by high academic performance. Moving away would thereby solve several problems at once, providing a better education and career, and possibly building new, more satisfactory interpersonal relationships and establishing healthy separation from parents.

Previous studies on Russian samples have shown that psychological predictors and correlates of internal and external migration intentions do not differ (Sychev et al., 2021); we focused on the opposite basic phenomenon — the desire to stay in the native region. We intended to explore two aspects. First, the direct effects of high school students’ satisfaction with significant interpersonal relationships on their intentions to stay in their native region and their well-being. Second, following the results of some recent studies (Gordeeva et al., 2023, Oriol et al., 2020), the mediation effects of prosocial life goals in this context. Thus, we formulated the main hypothesis of the study as follows: there would be a complex system of associations between migration intentions and adolescents’ significant interpersonal relationships (with family, school), and their prosocial life goals. This general hypothesis was divided into the following specific hypotheses:

H1. Satisfaction with relationships with teachers, school, and family would be an important predictor of high school students’ intentions to stay in their native region, and would be also related to their subjective well-being.
H2. The association of intention to stay in the home region with satisfaction with interpersonal relationships would be partially mediated by students’ prosocial life goals.
H3. The intention to stay to study and work in one’s own city/region (or low migration intentions) is associated with higher subjective well-being. However, when controlling for relationship factors such as satisfaction with school and teachers, family support, and community contribution life goals, this correlation would become insignificant.

The aim of the present study is to explore the relationship between the quality of significant interpersonal relationships, prosocial life goals, and low migration intentions of high school students, also considering the factors of academic performance and gender.

## Methods

### Participants and Procedure

The sample consisted of 5,635 16-18-year-old high-school students (10-11 grades) from 422 schools (*M* = 16.81, *SD* = .68), where 41% were males. 51% of respondents lived in cities and urban-type settlements of the Republic of Sakha (Yakutia); the rest lived in rural areas; all of them are 'uent in Russian. This study forms part of the All-Russian longitudinal project “Growing Together”. The online survey was conducted in the spring of 2022. The data collection procedure complies with the ethical standards of the Russian Psychological Society.

### Measures

*Intention to stay in the home region* was assessed through two statements: “I would like to study at the university in my hometown or my region”, and “After finishing university, I would like to work in my hometown, my region”. The degree of agreement with each item was assessed using the scale from 1 = *completely disagree* to 5 = *completely agree*. These statements were formulated taking into account the possible social desirability of students’ answers and also recent studies on place attachment (Boley et al., 2021), which complements the research on migration intentions and behavior. The scale that included these two items showed a high internal consistency, which indicates that the desire to leave the region to study is closely related to the desire to leave the region for a long time, not only for educational purposes, but also for residence. Cronbach’s alphas (α) for this and other scales used in the study are given in Table 1.

*Subjective well-being*. Three single-item scales were used to comprehensively measure well-being. Two questions were asked to assess happiness as one of the indicators of subjective well-being: “How do you usually feel?” and “How happy did you feel yesterday?”, rated on a scale of 0 (*very unhappy*) to 10 (*very happy*). The third, non-verbal scale (Andrews & Withey, 1976) was used to assess the participants’ attitude towards life. It included a series of seven symbolic faces with different emotional conditions: from cheerful and smiling to sad and very sad. The subjects were asked to choose the face corresponding to their attitude towards life. The reliability of the subjective well-being composite was .79.

*Satisfaction with school and teachers* was assessed by the corresponding subscales from the Life Satisfaction Scale (Huebner, 1994; Russian adaptation — Sychev et al., 2018), four items, and a non-verbal scale for assessing attitudes towards school, similar to the above-mentioned scale for assessing attitudes towards life (Andrews & Withey, 1976).

*Prosocial life goals*. To evaluate life goals related to community interests, helping other people, and general prosocial orientation, we used the Community Contribution Scale from the short version of the Aspiration Index questionnaire (Kasser & Ryan, 1993; Russian version — Gordeeva et al., 2023). This questionnaire includes various life goals presented as answers to the question: “How important is this goal to you?” Each of the options needs to be rated on a Likert scale from 1 (*not at all*) to 7 (*very important*). The Community Contribution Scale included the following three statements: “To work for the betterment of society”, “To help people in need”, “To help others improve their lives”.

*Social support*. We asked the following question to measure the degree of social support from family members: “If I have problems, I can go to…” with response options: Mom/Dad; Brother or sister; Other family members (grandparent(s), aunt, cousin, …) (Bruggeman et al., 2019). A five-point Likert scale was used, from 0 (*never*) to 4 (*always).*

*Grade point average (GPA), self-report.* To control the level of educational achievements, academic performance was assessed as the average of final grades for the last quarter in four main academic disciplines: algebra, geometry, Russian language, and literature.

### Data Analysis

Correlation analysis in the R free software environment for statistical computing and structural equation modeling (SEM) in the Mplus 8 program (Muthen & Muthen, 2015) were used to process the data. Due to the use of a categorical five-point response scale on the “intention to stay” items, modeling was carried out using the weighted least squares with mean and variance adjusted (WLSMV estimator). Standard errors were estimated considering the cluster composition of the sample associated with the distribution of students by schools. To assess the statistical significance of the mediated effects in the model, we ran bootstrap analysis (5,000 samples). Due to the large sample size, only results with a significance level of *p* < .001 were considered as suffciently significant and included in the interpretation.

## Results

The correlation analysis results (*Table 1*) indicate that intention to stay is related to all measured variables except age. Statistically significant correlations of intention to stay with satisfaction with school and teachers, social support of the family, community goals, and subjective well-being are positive and weak in magnitude (*r* from .13 to .25; all *p* < .001).

**Table 1:** Descriptive statistics and intercorrelations of measured variables (N = 5,635)

Measures	1	2	3	4	5	6	7	8	9	10	11	12	13	14
1. Intention to stay	–													
2. Happiness level usually	.19*	–												
3. Happiness level yesterday	.16*	.69*	–											
4. Positive attitude towards life	.13*	.55*	.45*	–										
5. Satisfaction with school	.25*	.43*	.36*	.38*	–									
6. Satisfaction with teachers	.22*	.38*	.32*	.30*	.68*	–								
7. Positive attitude towards school	.21*	.40*	.34*	.45*	.64*	.48*	–							
8. Community contribution goals	.20*	.27*	.23*	.25*	.36*	.37*	.31*	–						
9. Support from parents	.14*	.37*	.35*	.31*	.27*	.27*	.24*	.21*	–					
10. Support from brothers and sisters	.14*	.26*	.22*	.22*	.22*	.19*	.21*	.21*	.44*	–				
11. Support from other family members	.18*	.27*	.22*	.22*	.24*	.25*	.21*	.21*	.42*	.41*	–			
12. Grade point average	–.11*	.05*	.06*	.04	.13*	.14*	.14*	.07*	.13*	.04	.02	–		
13. Age (years old)	0	0	.01	–.01	.03	.05*	.02	.03	–.02	–.02	–.04	.01	–	
14. Gender (1 — male, 2 — female)	–.21*	–.15*	–.12*	–.10*	–.08*	–.11*	–.05*	.03	.02	.05*	–.09*	.25*	0	–
15. Residence (1 — rural, 2— urban)	–.07*	–.05*	–.03	–.07*	–.06*	–.01	–.07*	–.05*	–.03	–.06*	–.02	.02	0	.02
Cronbach’s α	.83	–	–	–	.81	.90	–	.79	–	–	–	.88	–	–
Means	2.46	6.61	6.63	5.48	3.2	3.47	4.89	5.05	3.14	2.58	2.2	4.05	16.72	–
Standard deviations	1.29	2.08	2.43	1.48	.89	.97	1.43	1.28	1.22	1.31	1.24	.59	1.08	–

Note. Significance: * p < .001, the column numbers correspond to the variable numbers in the rows.

The indicators of subjective well-being (the composite of happiness level usually and yesterday, and a positive attitude towards life) demonstrated the expected positive connections with each other, the magnitude of which ranges from moderate (*r* = .45; *p* < .001) to strong (*r* = .69; *p* < .001).

Positive attitude to school, satisfaction with school and relationships with teachers show similar correlations with each other: from moderate (*r* = .48; *p* < .001) to strong (*r* = .68; *p* < .001). Correlations between the indicators of subjective well-being and satisfaction with school and teachers, ranging from .30 (*p* < .001) to .45 (*p* < .001), are also moderate in magnitude.

Intercorrelations of indicators of social support from family members are close to each other and have a moderate value (*r* from .41 to .44; all *p* < .001). All indicators of social support are directly related to indicators of satisfaction with school and teachers, as well as subjective well-being (*r* from .22 to .37; all *p* < .001). The community contribution goals demonstrated positive correlations with all indicators of family support (*r* = .21; *p* < .001), subjective well-being (*r* from .23 to .27; all *p* < .001), and satisfaction with school and teachers (*r* from .31 to .37; all *p* < .001).

Male students have higher intention to stay (*M_male_* = 2.78, *SD* = 1.30, *M_female_* = 2.24, *SD* = 1.24, Welch’s t-test = 15.71, *df* = 4739, *p* < .001). Rural teenagers demonstrated a higher intention to stay compared to urban ones (*M_rural_* = 2.55, *SD* = 1.29, *M_urban_* = 2.37, *SD* = 1.29, Welch’s t-test = 5.38, *df* = 5626, *p* < .001).

To test the main hypothesis by means of SEM, we created a model where the intention to stay factor was dependent on all other factors and variables included in the model: satisfaction with school and teachers, family support, community contribution goals, subjective well-being, GPA, and gender. Subjective well-being was also considered as dependent on all other indicators. We assumed that the community contribution goals depended on satisfaction with school and teachers, family support, and gender, mediating the effect of those variables on intention to stay. Covariance between the factors of satisfaction with school and teachers, family support, and GPA, and their regression on gender, was allowed. Age and residence were not included in the model due to their weak effects on the dependent variables (less than 1% of the explained variance according to paired correlations). The resulting model (see *Figure*) showed a good fit to the data: χ^2^ = 1059.03, *df* = 133, *p* < .001, CFI = .950, TLI = .935, SRMR = .053, RMSEA = .035, 90% CI for RMSEA [.033, .037], PCLOSE = 1. Factor loadings for latent variables from this model are given in Table 2.

**Figure.**
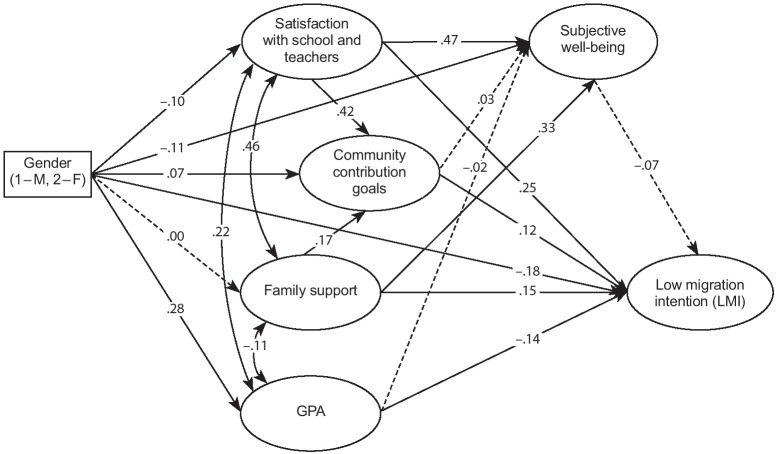
Structural model of the relationships of the measured variables with the intention to stay in the home region (residuals are omitted for parsimony; all coeffcients, with the exception of those located on the dotted lines, are significant at p < .001)

**Table 2:** Factor loadings of latent variables from the structure model

*Indicators*	Latent variables					
	Intention to stay	Subjective well-being	Community contribution	Satisfaction with school and teachers	Family support	Grade point average
Intention to stay, item 1	.99					
Intention to stay, item 2	.80					
Attitude towards life		.70				
Happiness level usually		.83				
Happiness level yesterday		.70				
Community contribution item 1			.76			
Community contribution item 2			.71			
Community contribution item 3			.76			
Satisfaction with teachers				.76		
Satisfaction with school				.84		
Positive attitude towards school				.74		
Support from parents					.74	
Support from brothers and sisters					.60	
Support from other family members					.61	
Grade in algebra						.79
Grade in geometry						.81
Grade in Russian language						.79
Grade in literature						.77

*Note. All factor loadings are significant at p < .001.*

The presented model (see *Figure*) demonstrates that intention to stay in the home region is positively related to satisfaction with school and teachers, family support, and community contribution goals. The negative correlation of intention to stay in the home region with GPA means that more successful high school students are more prone to migration intentions. The association with gender indicates that intention to stay in the home region is more typical for male students. At the same time, the path coeffcient from subjective well-being to intention to stay in the home region under the control of all these factors turned out to be negative and did not show the required level of statistical significance, although it was close (*p* = .009). Consequently, the positive paired correlation between intention to stay in the home region and subjective well-being is not reproduced in the model. This indicates that it is not only well-being that is important for intention to stay in the home region, but the factors that determine both well-being and intention to stay, taken together: satisfaction with school and teachers, family support, and gender.

Weak correlations of intention to stay with school satisfaction mediated through community contribution goals (standardized indirect effect = .05; *p* < .001), and with family support (.02; *p* < .001) are statistically significant. The total indirect effect of gender on intention to stay through the factors of school satisfaction, community goals, and school performance is also statistically significant (–.04; *p* < .001). Thus, the results demonstrate that in high school students, the most influential psychological factors of intention to stay in the home region are community contribution goals, satisfaction with school and teachers, and family support, as well as gender and GPA.

## Discussion

These results confirmed the hypotheses and indicated that satisfaction with relationships in the family, with teachers, and with school life significantly reduced migration intentions of young people. It was found that their satisfaction with the school and gratifying relationships, e.g., with teachers and parents, represent the important factors for both subjective well-being and the absence of migration intentions, the desire to stay in the home region. In other words, high school students who highly appreciate the quality of their relationship with their teachers, receive their acceptance and support, have a positive attitude towards school, and experience greater support from their family members feel happier (which confirms previous studies, Gordeeva & Sychev, 2023; Vansteenkiste & Ryan, 2013). Those students also tend to set prosocial goals, which implies a desire to help other people. Altogether, these factors contribute to the students’ desire to stay in their home region/city.

This is the first time that a study has confirmed the association of the quality of relationships with those in the immediate environment in shaping high school students’ migration intentions, and their desire to stay or to leave the region for further education and work. At the same time, the results fall within the scope of research focused on the significance of perceptions of the broader social environment and community in shaping migration intentions (Cai et al., 2014), the role of relatives and social ties abroad (Efremenkova et al., 2023), and the role of parents and friends as a factor in migration (Frieze et al., 2011). This direction of research can be developed through a more detailed analysis of the role of three types of significant social relationships — with parents, peers, and teachers — as to a greater or lesser extent satisfying the basic psychological needs for autonomy, competence, and relatedness in the formation of migration intentions.

Following the research on the role of educational level (Docquier et al., 2014; Graham & Markowitz, 2011) in migratory intentions, we have now gained evidence on the influence of academic achievement in school (GPA) on migratory intentions. As expected, more successful students had more pronounced intentions to leave the region. But our data allow the suggestion that the propensity of more educated individuals to manifest their desire to move abroad may be associated not only with the fact that they hope to find a well-paid job, but also with seeking more satisfying personal relationships. According to our previous studies, migration intentions of young people are associated with high introjected motivation (Rudnova et al., 2023), and at the same time, with higher academic achievements. However, we can suggest that parental pressure and an authoritarian, autonomy-frustrating parenting style might often be the hidden reason for these achievements, and therefore, young people seek to avoid them by leaving their parental family and moving to another city/ region.

Consistent with previous research on the relationship between subjective wellbeing and migration intentions discussed above (Hendriks & Burger, 2020), our data indicate a similar pairwise direct correlation. At the same time, our hypothesis about the conditionality of this relationship by several other psychological variables also found confirmation: when controlling for psychological variables such as satisfaction with relationships at school and in the family, the link between subjective well-being and migration intentions became insignificant.

Regarding gender, we found that young men demonstrated a greater desire to stay in the region/ city, which again, was consistent with the outcome of previous studies on Russian samples of the intentions of internal migration (Sychev et al., 2021). Interestingly, the somewhat greater propensity of young women to form migration intentions, accompanied by their higher academic achievements and less satisfaction with school and teachers, to some extent confirms the phenomenon of “frustrated achievers” prone to trying their luck in other countries and regions (Graham & Markowitz, 2011). Probably there are other factors that induce female respondents to form migratory intentions, as evidenced by the different nature of migratory motives for males and females (De Jong et al., 1996).

## Conclusions

Migration intentions of young people form a complex research and practical problem that requires a systematic approach to be fully understood. Based on the data collected on a sample of Russian high school students from Yakutia region, our study found that the important psychological factors reducing high school students’ migration intentions were satisfaction with school and teachers, family support, and prosocial life goals. These data are definitely of a certain practical significance, since they indicate a possibility to influence migration processes in the regions. Potentially, to some extent, that can be achieved through work with school teachers and providing psychological support to families. Also, male gender and relatively low academic performance at school were important contributors to the intention to stay in one’s home region.

## Limitations and Future Directions

The correlational design that we used does not allow testing the hypothesis of a causal relationship between the migration intentions and other psychological variables in the study. Further research applying a more rigorous design might include testing a hypothesis that dysfunctional and non-supportive relationships in the family and school negatively influence the formation of prosocial life goals, and indirectly affect adolescents’ migration intentions. Other possible factors to be included in future studies are the quality of relationships with peers, the feeling of being accepted or consistently rejected for some reason, as well as the number of online and offline friends. Potentially, these indicators could also play a role in one’s intention to stay in the home region/ country or to leave it.
